# The concerted actions of microRNA-29a and interferon-β modulate complete Freund's adjuvant-induced inflammatory pain by regulating the expression of type 1 interferon receptor, interferon-stimulated gene 15, and p-extracellular signal-regulated kinase

**DOI:** 10.1016/j.bjao.2024.100376

**Published:** 2025-02-03

**Authors:** Chien-Cheng Liu, Kuo-Chuan Hung, Yu-Yu Li, Eagle Yi-Kung Huang, Chin-Chen Chu, Lok-Hi Chow, Ping-Heng Tan

**Affiliations:** 1 Department of Anesthesiology, E-Da Hospital, I-Shou University, Kaohsiung, Taiwan; 2 Department of Anesthesiology, Chi Mei Medical Center, Tainan, Taiwan; 3 School of Medicine, College of Medicine, National Sun Yat-sen University, Kaohsiung, Taiwan; 4 Department of Anesthesiology, Chi Mei Hospital, Chiali, Tainan, Taiwan; 5 Department of Pharmacology, National Defense Medical Center, Taipei, Taiwan; 6 Department of Anesthesiology, Taipei Veterans General Hospital, Taipei, Taiwan; 7 National Yang-Ming Chiao-Tung University, School of Medicine, Taipei, Taiwan

**Keywords:** IFN-β, inflammatory pain, interferon-stimulated gene 15, microRNA 29a, type 1 interferon receptor

## Abstract

**Background:**

Previous research has shown that type 1 interferons (IFN), such as IFN-α and IFN-ß, possess antiviral and antinociception effects. Elevated levels of microRNA-29a (miR-29a) have been observed during inflammatory pain, and as miR-29a targets the type 1 IFN receptor (IFNR1), our study aimed to investigate the involvement of miR-29a, type 1 IFN, and IFNR1 in inflammatory pain.

**Methods:**

Inflammatory pain was induced in male rats using complete Freund's adjuvant (CFA). The changes in miR-29a, IFN-ß, and IFNR1 were measured on Days 2, 3, 5, 7, and 10 post-CFA injection and expression of IFNR1, phospho-ERK (phosphorylated extracellular signal-regulated kinase) (p-ERK), extracellular signal-regulated kinase (ERK), and IFN-stimulated gene 15 (ISG15) were measured in rats that received an miR-29a inhibitor or miR-29a mimic.

**Results:**

Our results demonstrated elevated miR-29a expression (CFA 3 days: mean difference [95% confidence interval, CI]: 0.860 [0.657–1.062]; CFA 5 days: mean difference [95% CI]: 1.120 [0.917–1.322], *P*<0.001, *n*=6) and decreased IFNR1 expression (CFA 3 days: mean difference [95% CI]: −0.300 [−0.470 to −0.130]; CFA 5 days: mean difference [95% CI]: −0.330 [−0.515 to −0.145], *P*=0.004, *n*=6) from Days 3–5 post-CFA induction, with IFN-ß expression showing a significant increase from Day 2 (F [3.30, 16.5]=34.3 for factor time, *P*≤0.01, *n*=6). Treatment with an miR-29a inhibitor alleviated CFA-induced mechanical allodynia and thermal hyperalgesia by Day 5 (*P*<0.001, *n*=9), concomitant with upregulation of IFNR1 and ISG15 expression, and downregulation of p-ERK (IFNR1; CFA 5 days + miR-29a inhibitor *vs* CFA 5 days; mean difference [95% CI]: 30.00 [20.31–39.69]; ISG15 conjugates; CFA 5 days + miR-29a inhibitor *vs* CFA 5 days, mean difference [95% CI]: 1.000 [0.9144–1.086]; free ISG15, mean difference [95% CI]: 2.402 [2.171–2.633]; p-ERK; CFA 5 days + miR-29a inhibitor *vs* CFA 5 days, mean difference [95% CI]: −32.00 [−34.10 to −29.90], *P*<0.001, *n*=9). Furthermore, in naïve rats, administration of an miR-29a mimic-induced mechanical allodynia, which was reversed by an ERK antagonist (*P*<0.001, *n*=6), associated with decreased IFNR1 and increased p-ERK expression (IFNR1; miR-29a mimic + dimethyl sulfoxide *vs* naïve; mean difference [95% CI]: −57.00 [−65.78 to −48.22]; miR-29a mimic + ASN007 *vs* naïve; mean difference [95% CI]: −60.00 [−71.00 to −49.00]. p-ERK; miR-29a mimic + dimethyl sulfoxide *vs* naïve, mean difference [95% CI]: 52.00 [47.01–56.99]; miR-29a mimic + ASN007 *vs* naïve, mean difference [95% CI]: 47.00 [42.51–51.49]; *P*<0.001, *n*=6).

**Conclusions:**

Inhibiting miR-29a expression attenuates inflammatory pain by modulating IFNR1, ISG15, and p-ERK expression, highlighting the interactive roles of miR-29a and IFN-ß in the regulation of inflammatory pain.

Type I interferons (IFN), such as α-IFN, β-IFN, ω-IFN, and τ-IFN, share receptors and pathways,^1^^,^^2^ regulating immune surveillance against pathogens and malignancies in the immune, endocrine, and central nervous systems.^3^^,^^4^ Produced by CNS cells such as microglia, astrocytes, and neurones,^5^^,^^6^ IFN influence neuronal function and may cause neuropsychiatric symptoms.^7^^,^^8^

IFN, notably IFN-β, are used to treat infections, neoplasms, and autoimmune diseases because of their immunomodulatory and antiviral properties.^7^^,^^8^ IFN-β, binding to type 1 IFN receptor (IFNR1), modulates immune responses and protects neurones from neurotoxic and inflammatory damage.^9^ Recently, IFN-β induced by small intestinal commensal bacteria was reported to exert an anti-inflammatory effect and protect against experimental colitis.^10^ IFN-β therapy alters microRNA (miRNA) expression profiles in multiple sclerosis patients, including downregulation of microRNA-29a (miR-29a) and miR-29c.^11^ miR29 expression was shown to be increased early after IFN-β treatment but decreased again by 48 h.^12^ This suggests a negative feedback mechanism through miR-29a, limiting type 1 IFN sensitivity to prevent overactivation or sustained responses.

Recent studies emphasise the crucial role of neurone–glia interactions, particularly those involving microglia and astrocytes, in pain pathogenesis through the release of proinflammatory mediators.^13^ While IFN-γ has been linked to the development of neuropathic pain through these mechanisms,^14^ the effects of IFN-β on spinal cord pain sensitivity remain less clear. Our miRNA microarray analysis identified a significant increase in miR-29a expression during inflammatory pain, with miR-29a targeting IFNR1.^15^ This study aimed to clarify the roles of IFN-β, miR-29a, and IFNR1 in modulating nociceptive responses by examining their expression in the spinal cord during inflammatory pain. Additionally, we explored the involvement of IFN-stimulated gene 15 (ISG15), a key component of the type 1 IFN pathway,^16^^,^^17^ through its induction by type 1 IFN.

## Methods

### Animals

The experiments followed the Ethical Guidelines of the International Association for the Study of Pain^18^ and ARRIVE guidelines.^19^ We utilised male Sprague Dawley rats (6–8 weeks old, 250–350 g) sourced from the National Laboratory Animal Center, Taipei, Taiwan. Two rats were housed per cage, with a 24-h acclimatisation period in the experimental room. Rats had *ad libitum* access to water and standard laboratory diet. The room temperature was maintained at 23°C, with a 12-h daylight/dark cycle. Institutional review board approval (AUP- 105-57-02) from I-Shou University, Kaohsiung, Taiwan was obtained for all animal procedures. A trained member of staff, blinded to study group allocation, performed the behavioural tests and dissected the spinal cord.

### Drugs and administration

Polyethyleneimine (PEI) (Fermentas, Inc., Glen Burnie, MD, USA) was used without purification. Rat miR-29a-3p mimic (miRNA mimic, rno-miR-29a-3p sequence ‘UAGCACCAUCUGAAAUCGGUUA’, C-320321-05, Dharmacon Inc., Lafayette, CO, USA), inhibitor, and scrambled control (miRNA mimic negative control, CN-001000-01; Dharmacon) were used. miRs were mixed with PEI at room temperature for 10 min before intrathecal (i.t.) delivery to enhance penetration and reduce degradation. The ratio used was 1 μl of PEI 100 mM solution per 5 μg of miRNA. miRNA solutions were diluted in water with dextrose 5% for 10 min before injection. Drugs (2 or 4 nmol) were administered i.t. The ERK inhibitor ASN 007 (MedChemExpress, Monmouth Junction, NJ, USA), or vehicle (20% dimethyl sulfoxide [DMSO]) was injected i.t. ASN 007 was dissolved in DMSO 20% to a final concentration of 1 μg μl^−1^.^20^

Each i.t. injection was done under brief sevoflurane anaesthesia using a 30 G needle between L5 and L6 to deliver the reagents (40 ul) into the cerebrospinal fluid. Immediately after the needle entry into the subarachnoid space (change in resistance), a brisk tail-flick was observed.^21^

### Luciferase reporter assay

The luciferase reporter assay was designed to investigate whether miR-29a can post-transcriptionally modulate IFNAR1 expression in HEK293 cells. To achieve this, a 212 bp fragment of the IFNAR1 3′UTR, which contains the miR-29a-3p binding site, was cloned into the pmirGLO dual-luciferase miRNA target expression vector. The cloning was performed using restriction enzymes XbaI and SacI, and the vector was verified by sequencing.

#### Cell culture and transfection

HEK293 cells were seeded at a density of 2.5 × 10⁵ cells per well in 24-well plates, using Dulbecco's Modified Eagle Medium supplemented with fetal bovine serum FBS 10%, penicillin 10 U ml^−1^, streptomycin 10 μg ml^−1^, glutamine 2 mM, and sodium pyruvate 1 mM. The following day, the cells were co-transfected with the pmirGLO-IFNAR1-3′UTR vector 0.5 μg and either miR-29a-3p mimics (C-320321-05, Dharmacon) or a negative control (miR-NC, CN-001000-01, Dharmacon), 50 nM or 100 nM.

#### Luciferase assay and data analysis

**Fig 1:**
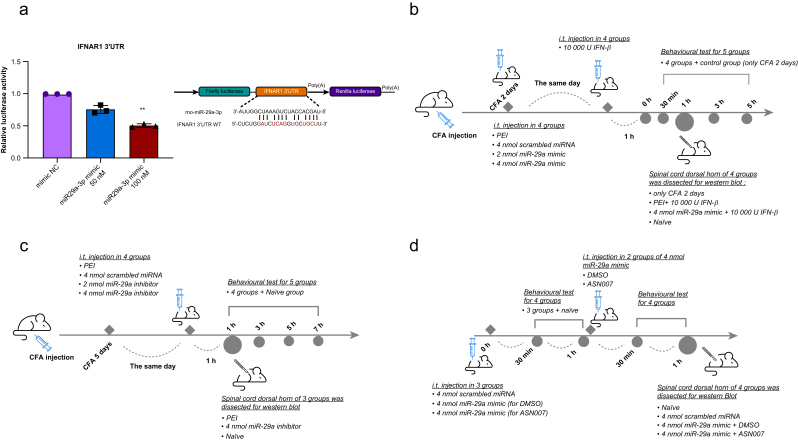
Luciferase reporter assay for miR-29a and IFNR1 and flow chart showing the experimental design of the animal studies. (a) Genomic location and structure of a luciferase reporter based on IFNR1 (pmirGLO-IFNAR1 3′UTR vector) containing an miR-29a-3p binding motif. The miR-29a-3p mimic (100 nM) appeared to reduce luciferase activity in HEK293 cells after cotransfection with the IFNR1-luciferase reporter construct, as shown by the decrease in normalised luciferase activity compared with the mimic NC control (*n*=3, individual data points shown). (b) Examination of the effects of IFN-β and the miR-29a mimic on the behaviour of rats 2 days after CFA injection. (c) Examination of the effect of the miR-29a inhibitor on pain-related behaviour in rats 5 days after CFA injection. (d) Examination of the attenuation of miR-29a-induced mechanical hyperalgesia by a p-ERK antagonist in naïve rats. CFA, complete Freund's adjuvant; DMSO, dimethyl sulfoxide; IFNAR1 3′UTR, interferon Alpha/Beta receptor 1 3′ untranslated region; IFN-β, interferon-β; miR-29a-3p, microRNA-29a-3p; miRNA, microRNA; mo-miR, mouse-microRNA; NC, negative control; PEI, polyethyleneimine; WT, wild type. ∗∗*P*=0.003, miR-29a-3p mimic *vs* mimic NC.

After 24 h of incubation, the cells were lysed using passive lysis buffer. The luciferase activity was then measured using the Dual-Luciferase Reporter Assay System (E1910, Promega, Madison, WI, USA). In this system, firefly luciferase activity, which reflects the regulation of the IFNAR1 3′UTR by miR-29a-3p, was normalised to Renilla luciferase activity, which serves as an internal control for transfection efficiency and cell viability. The ratio of firefly to Renilla luminescence was calculated for each condition. A decrease in this ratio in the presence of miR-29a-3p compared with the negative control would indicate that miR-29a-3p represses IFNAR1 expression. The experiment was conducted in triplicate to ensure statistical reliability (Fig. 1a).

### Examination of the changes in miR-29a, IFN-β, and IFNR1 expression during complete Freund's adjuvant-induced inflammatory pain over time

Thirty microliters of complete Freund's adjuvant (CFA) was injected into the left hind paws of the rats in each experimental group (*n*=6/group) to establish inflammatory pain, whereas a group of naïve rats did not receive an CFA injection (*n*=7/group). The five groups of rats were examined separately for mechanical allodynia on the 2nd, 3rd, 5th, 7th, and 10th days after CFA injection. The rats were killed by decapitation under deep anaesthesia (i.p. pentobarbital 120 mg kg^−1^). The spinal cord dorsal horns of the rats in each group were dissected after the behavioural tests to measure miR-29a, IFN-β, and IFNR1 levels via western blotting.

### Evaluation of the effects of IFN-β and the miR-29a mimic on pain-related behaviour 2 days after CFA injection

To investigate the effect of IFN-β on inflammatory pain, four groups of rats (*n*=8/group) were used. The naïve group received an injection of saline 30 μl. The other three groups were injected i.t. with IFN-β 1000 U, 3000 U, or 10000 U 2 days after the injection of CFA. Mechanical allodynia was assessed using von Frey filaments at 30 min, 1 h, 3 h, and 5 h after IFN-β injection. To evaluate the effect of the miR-29a mimic, IFN-β 10 000 U was used. Four groups of rats were treated 2 days after CFA injection with either a transfection agent (PEI), scrambled miRNA 4 nmol, miR-29a mimic 2 nmol, miR-29a mimic 4 nmol. A separate group that received only CFA served as the control. One hour after i.t. injection of PEI, scrambled miRNA, or the miR-29a mimic, all groups were injected with IFN-β 10 000 U (*n*=8/group). Mechanical allodynia and thermal hyperalgesia were then measured at 30 min, 1 h, 3 h, and 5 h post–IFN–β injection.

At the end of the experiment, the rats were killed by decapitation under deep anaesthesia (i.p. pentobarbital 120 mg kg^−1^). To investigate the mechanisms of IFN-β and the miR-29a mimic, spinal cord dorsal horns were dissected from four additional groups of rats: naïve rats (receiving no substances or CFA), rats that received only CFA, rats treated with PEI and IFN-β 10 000 U (PEI administered 1 h before IFN-β), and rats treated with miR-29a mimic 4 nmol and IFN-β 10 000 U (miR-29a mimic administered 1 h before IFN-β). These tissues were collected for Western blot analysis of IFNR1, phospho-ERK (p-ERK), ERK, and ISG15, 1 h after IFN-β treatment on Day 2 after CFA injection (*n*=8/group) (Fig. 1b).

### Evaluation of the effects of the miR-29a inhibitor on pain-related behaviour 5 days after CFA injection

To investigate the effect of the miR-29a inhibitor on inflammatory pain, four groups of rats were treated with a transfection agent (PEI), scrambled miRNA 4 nmol, the miR-29a inhibitor 2 nmol or the miR-29a inhibitor 4 nmol (*n*=9/group) 5 days after CFA injection (Fig. 1c). A naïve rat group that received only an CFA injection for 5 days was included as the control group. Mechanical allodynia and thermal hyperalgesia were assessed at 1, 3, 5, and 7 h after the injection of PEI, scrambled miRNA, or the miR-29a inhibitor either 2 or 4 nmol. At the end of the experiment, the rats were killed by decapitation under deep anaesthesia (i.p. pentobarbital, 120 mg kg^−1^). To investigate the mechanism of action of the miR-29a inhibitor, spinal cord dorsal horns were dissected from three groups of rats: naïve rats (not receiving any substance or CFA), rats that received PEI injection, and rats that received the miR-29a inhibitor 4 nmol. These tissues were collected 1 h after the injection of PEI or the miR-29a inhibitor for Western blot analysis of IFNR1, p-ERK, ERK, and ISG15 (*n*=9/group) on Day 5 after CFA injection.

### Examination of the attenuation of miR-29a-induced mechanical hyperalgesia by a p-ERK antagonist in naïve rats

To further investigate whether the effect of miR-29a results from the upregulation of p-ERK, an antagonist of p-ERK was used to antagonise miR-29a mimic-induced mechanical hyperalgesia in naïve rats (Fig. 1d). Two groups of rats were treated with the miR-29a mimic 4 nmol followed by injection of DMSO or the p-ERK antagonist (ASN007) 30 μg, 1 h later. The control groups included naïve rats and rats that received an i.t. injection of scrambled miR-29a 4 nmol (*n*=6/group). The von Frey test was performed on all four groups every 30 min for 1 h. After the behavioural test, the spinal cord dorsal horns of the four groups were dissected for Western blot analysis of p-ERK, ERK, and IFNR1. The rats were killed by decapitation under deep anaesthesia (i.p. pentobarbital 120 mg kg^−1^).

### Behavioural tests

#### The von Frey test

To assess mechanical hypersensitivity and the development of allodynia, the hind paw withdrawal threshold in response to von Frey filament stimulation was measured using the up-and-down method as described by Chaplan and colleagues.^22^ Initially, the animals were allowed to acclimatise to the environment by placing them in individual clear Plexiglas boxes on an elevated wire mesh platform for 30 min. Subsequently, a series of von Frey filaments (0.02, 0.07, 0.16, 0.4, 0.6, 1.0, 1.4, and 26 g; Stoelting Co., Wood Dale, IL, USA) were applied perpendicularly to the plantar surface of each hind paw for 3–5 s per filament. The test began with the application of a 0.6 g filament. A positive response, defined as a clear paw withdrawal or shaking, prompted the application of the next lower filament, while a negative response led to the application of the next higher filament. The testing involved five stimuli, and the pattern of responses was converted to a 50% paw withdrawal threshold using Dixon's up-and-down method.^23^

#### The plantar test

Thermal hypersensitivity was assessed using the plantar test, following the Hargreaves method.^24^ After acclimatising the animals to their environment on a glass plate surface, the mid-plantar surface of the hind paw was exposed to a radiant heat source through a glass floor using the Hargreaves apparatus (Stoelting Co.). The heat intensity was adjusted to produce a baseline withdrawal latency of ∼12–15 s in control animals, with a maximum cut-off time of 20 s to prevent tissue damage. The stimulus was terminated either upon paw withdrawal or after reaching the cut-off time. To avoid thermal sensitisation and behavioural disruption, three trials were conducted at 5-min intervals. The mean paw withdrawal latency from these trials was used for statistical analysis.

### RNA extraction and TaqMan miRNA assays

The miRNA concentration was measured with Thermo Fisher's TaqMan microRNA assay according to the manufacturer's instructions. For reverse transcription of miR-29a-3p and 4.5S, TaqMan microRNA assays were used, which included reverse transcription primers for each miRNA. Quantitative polymerase chain reaction (qPCR) was conducted using primers and probes provided by TaqMan Universal Master Mix II, no UNG (4440047, Applied Biosystems, Förster City, CA, USA).

### Western blot analysis

A 1:20 ratio of Tissue Protein Extraction Reagent to protease inhibitors (PIERCE, Rockford, IL, USA) was used to extract protein from spinal cord dorsal horns, followed by homogenisation and centrifugation. Protein concentration was assessed using a Bio-Rad Protein Assay Kit (Bio-Rad Laboratories, Hercules, CA, USA), and proteins were stored at −20°C. Electrophoresis separated 30 μg of total protein on a 10% SDS-PAGE gel, transferred to PVDF membranes, and blocked with TBS-T buffer and 5% non-fat milk. Primary antibodies against IFNAR1 (1:1000; A1715; ABclonal, Wuhan, China), pErk1/2 (1:1000; Cell Signaling Technology, Danvers, MA, USA), anti-ISG15 (1:500; A2416; ABclonal, USA), and Erk1/2 (1:1000; 137F5; Cell Signaling Technology) were added and incubated overnight at 4°C in fresh blocking buffer. As a loading control, the blots were incubated with GAPDH (1:16 000, MA5-15738, Thermo Fisher, Waltham, MA, USA) and b-actin (1:20 000, A5441, Sigma-Aldrich, St. Louis, MO, USA). After the membranes were washed for 30 min at room temperature with washing buffer, the membranes were incubated for 1.5 h at room temperature with horseradish peroxidase-labelled goat anti-rabbit IgG (1:5000, 20202, Leadgene Biomedical, Tainan City, Taiwan) or mouse anti-IgG (1:5000, 20102). Chemiluminescence Reagent Plus (Millipore, Billerica, MA, USA) was used to visualise the bands and the molecular weights of the bands were determined using an enhanced chemiluminescence (ECL) kit (1705061, Bio-Rad). ImageJ software (NIH, Bethesda, MD, USA) was used to analyse the intensity of the selected bands.

### Statistical analysis

GraphPad Prism 8.0 (GraphPad Software, La Jolla, CA, USA) was used for the statistical analyses. All of the data are expressed as mean difference [95% confidence interval]. The sample sizes used in each experiment were based on our previous studies.^15^^,^^25^ We anticipated significant increases in paw withdrawal thresholds and latency for 6 h after i.t. injection of the miR-29a mimic. The mean (standard deviation) paw withdrawal threshold was 6.0 (1.2), and the expression of the IFNR1 decreased by a mean (standard deviation) of 40% (0.8) after the miR-29a mimic injection. A power analysis using data from these studies was performed to estimate the minimum number of animals needed to detect significant differences with 80% power and a significance level of 0.05. The analysis indicated that, with the observed effect sizes, a sample size of five to 10 per group would be sufficient to detect meaningful differences in paw withdrawal thresholds and receptor expression changes. Two-way repeated measures (RM) analysis of variance (anova) and *post hoc* Bonferroni correction were used to analyse the behavioural data after the i.t. injection of the miR-29a mimic or inhibitor. One-way RM anova followed by a *post hoc* Bonferroni correction was used to analyse the behavioural test, quantitative reverse transcription‒PCR, Western blot analysis, and luciferase reporter assay results after i.t. injection of the p-ERK antagonist ASN 007. A *P*-value <0.05 was considered to indicate statistical significance.

## Results

### Luciferase assay for miR-29a and IFNR1

Firefly luciferase activity showed a decrease in pmirGLO-IFNAR1-3′UTR-transfected cells treated with the miR-29a-3p mimic. Specifically, there was a reduction of ∼24% after administering a 50 nM dose and a reduction of about 48% after a 100 nM dose, compared with negative control (NC) cells (Fig. 1a). These observations suggest that miR-29a-3p may contribute to the downregulation of IFNR1 expression, potentially targeting IFNR1 directly.

### Examination of the temporal changes in miR-29a, IFN-β, and IFNR1 expression during CFA-induced inflammatory pain

**Fig 2:**
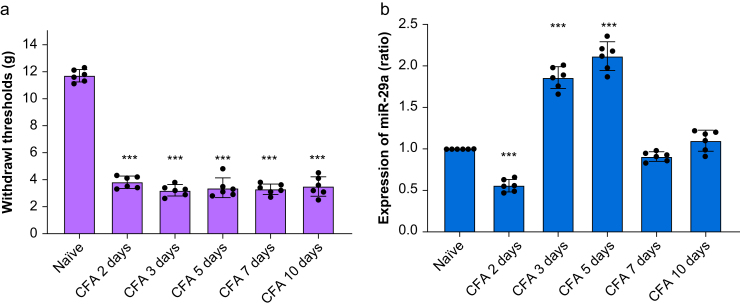
Behaviour and temporal changes in miR-29a expression after CFA injection. (a) A significantly decreased mechanical paw withdrawal threshold was observed after CFA injection. Individual data points are shown alongside bars representing group means. ∗∗∗*P*<0.001 *vs* naïve; one-way repeated measures analysis of variance (RM anova) with Bonferroni *post hoc* correction; *n*=7 (naïve group), *n*=6 (CFA groups). (b) MiR-29a expression was significantly reduced on the 2nd day, followed by an increase from the 3rd to the 5th day after CFA injection. Individual values are displayed alongside the mean (standard deviation), with bars providing an overview of the group mean. ∗∗∗*P*<0.001 *vs* naïve; one-way RM anova with Bonferroni *post hoc* correction; *n*=6/group. CFA, complete Freund's adjuvant; miR-29a, microRNA-29a.

**Fig. S1:**
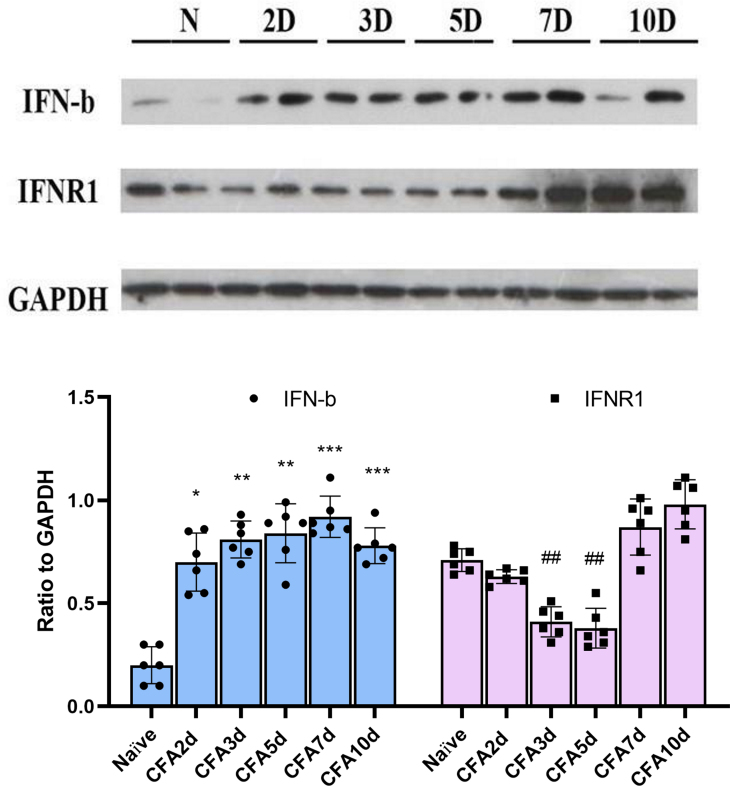


To study the temporal changes in the IFNR1, IFN-β, and miR-29a levels, inflammatory pain was induced by intradermal injection of CFA. Injection of CFA into the hind paw significantly decreased the paw withdrawal threshold on Days 2–10 (*P*<0.001; Fig. 2a), suggesting the development of inflammatory pain. MiR-29a was significantly downregulated on Day 2 and upregulated on Days 3 and 5 after CFA injection (Fig. 2b). IFN-β was upregulated during the Day 2–10 period after CFA injection (*P*<0.001; Supplementary Fig. S1). Type 1 IFNRs were significantly downregulated at Days 3–5 after CFA injection (Supplementary Fig. S1).

### Evaluation of the effects of IFN-β and the miR-29a mimic on pain-related behaviour 2 days after CFA injection

**Fig 3:**
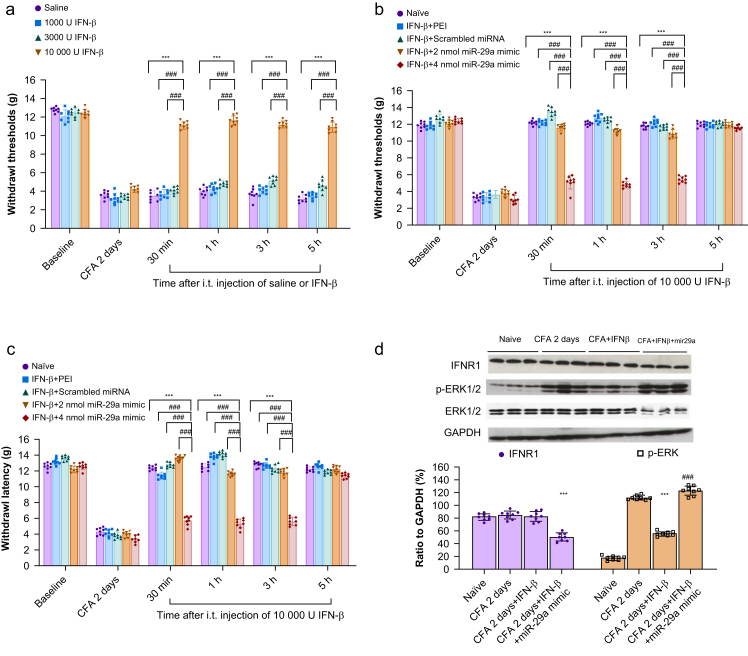
Behavioural test results and evaluation of type 1 interferon receptor (IFNR1) and p-ERK expression in the spinal cord of CFA-injected rats receiving intrathecal (i.t.) injection of interferon-β with or without miR-29a mimic 4 nmol. (a) Two days after CFA injection, four groups of rats received i.t. injections of IFN-β (1000 U, 3000 U, or 10 000 U) or an equivalent volume of saline. Mechanical allodynia was assessed at 0.5, 1, 3, and 5 h post-treatment using the paw withdrawal test. Rats treated with 10 000 U IFN-β exhibited significant inhibition of CFA-induced mechanical allodynia from 0.5 h to 5 h. ∗∗∗*P*<0.001, IFN-β 10 000 U *vs* saline; ###*P*<0.001, IFN-β 10 000 U *vs* 3000 U and IFN-β 1000 U; two-way repeated measures analysis of variance (RM anova) with Bonferroni *post hoc* correction, *n*=8/group. Individual data points are shown along with bar charts to indicate the distribution of the data. (b, c) I.T. injection of miR-29a mimic 4 nmol ml^−1^ abrogated the anti-allodynia effect of IFN-β 10 000 U for 3 h on the 2nd day after CFA injection. ∗∗∗*P*<0.001, miR-29a mimic 4 nmol *vs* naïve; ###*P*<0.001, miR-29a mimic 4 nmol *vs* PEI, scrambled miRNA, and miR-29a mimic 2 nmol; two-way RM anova with Bonferroni *post hoc* correction, *n*=8/group. Individual data points are presented alongside bars for clarity. (d) I.T. injections of miR-29a mimic 4 nmol with IFN-β reduced IFNR1 expression and reversed the inhibition of p-ERK induced by 10 000 U IFN-β 2 days after CFA injection. IFNR1: ∗∗∗*P*<0.001, CFA 2 days + IFN-β + miR-29a mimic *vs* CFA 2 days; p-ERK: ###*P*<0.001, CFA 2 days + IFN-β + miR-29a mimic *vs* CFA 2 days + IFN-β; one-way RM anova with Bonferroni *post hoc* correction, *n*=8/group. Individual data points are displayed along with bars to represent the mean (standard deviation). Original Western blot images are provided in the Supplementary material. CFA, complete Freund's adjuvant; GADPH, glyceraldehyde-3-phosphate dehydrogenase; IFN-β, interferon-β; miRNA, microRNA; p-ERK, phospho-ERK; PEI, polyethyleneimine.

Among the doses of IFN-β of 1000 U, 3000 U, and 10 000 U, the i.t. injection of 10 000 U significantly attenuated the reduction in the paw withdrawal threshold for at least 5 h on the 2nd day after CFA injection (Fig. 3a). I.t. injection of miR-29a mimic 4 nmol but not 2 nmol on the 2nd day after CFA injection could abrogate the increase of mechanical withdrawal threshold (Fig. 3b) and thermal withdrawal latency induced by injection of 10 000 U IFN-β for 5 h (Fig. 3c).

**Fig. S2:**
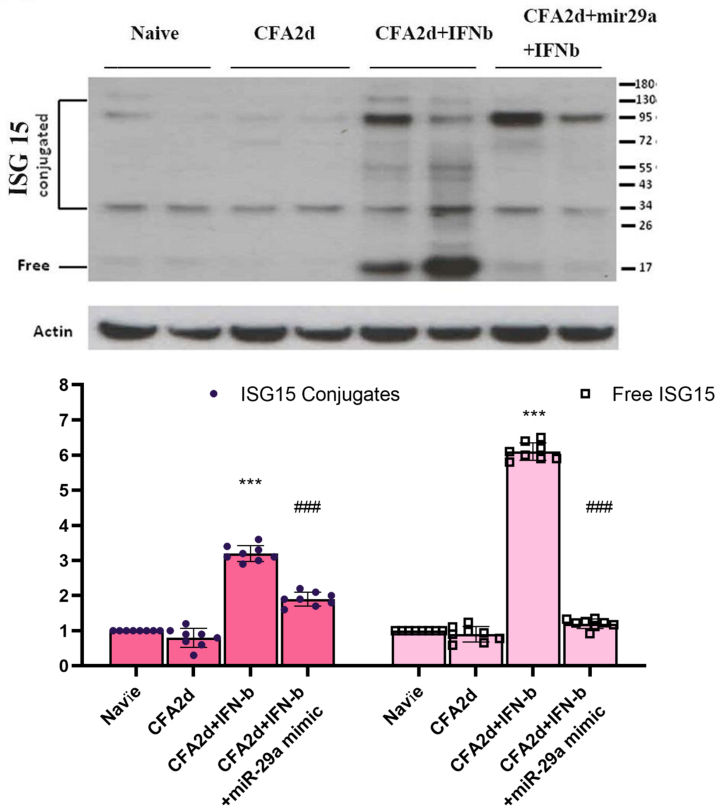


Simultaneously, significant downregulation of IFNR1 was noted after injection of IFN-β 10000 U and the miR-29a mimic 4 nmol (Fig. 3d). Significant upregulation of ISG15 and downregulation of p-ERK were noted 1 h after injection of IFN-β 10000 U (Fig. 3d, Supplementary Fig. S2). In contrast, significant decreases in conjugate and free ISG15 levels and increases in p-ERK levels were detected after the injection of IFN-β 10000 U and miR-29a mimic 4 nmol (Fig. 3d, Supplementary Fig. S2).

### Evaluation of the effect of the miR-29a inhibitor on pain-related behaviour 5 days after CFA injection

**Fig 4:**
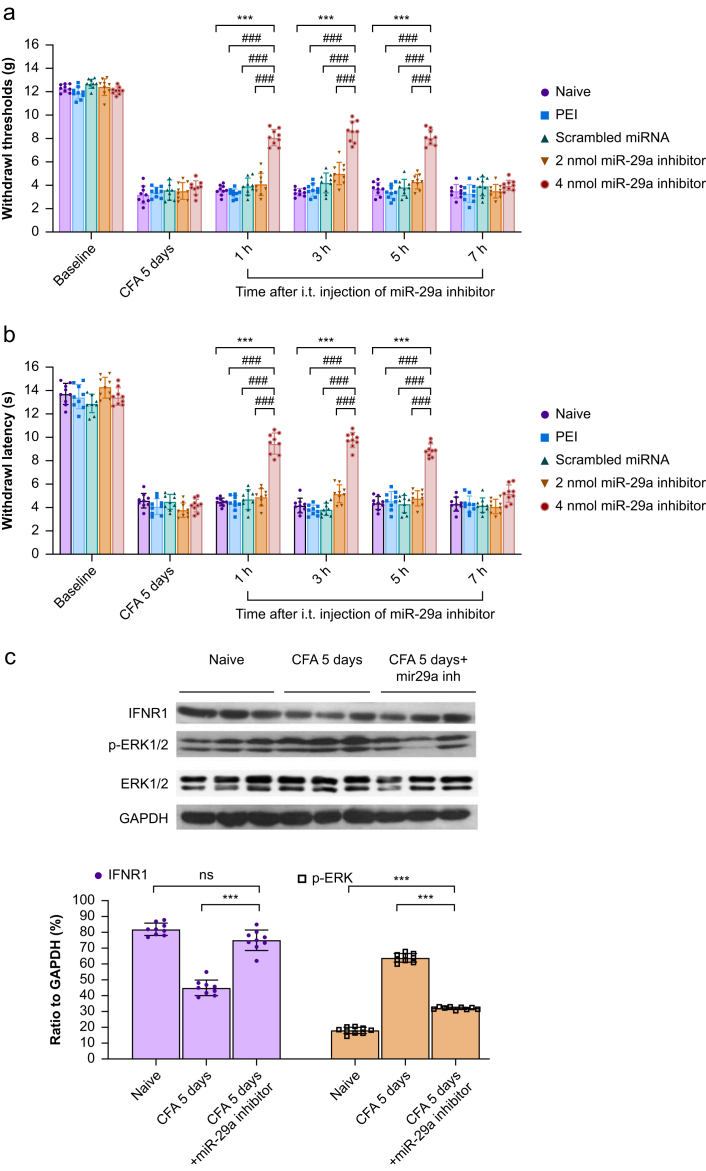
Behavioural response and expression of type 1 interferon receptor (IFNR1) and p-ERK in the spinal cord of rats after i.t. injection of miR-29a inhibitor 4 nmol 5 days after CFA injection. (a, b) Five days after CFA injection, i.t. administration of miR-29a inhibitor 4 nmol significantly increased paw withdrawal threshold and thermal withdrawal latency for 5 h in a dose-dependent manner. ∗∗∗*P*<0.001, miR-29a inhibitor 4 nmol *vs* naïve; ###*P*<0.001, miR-29a inhibitor 4 nmol *vs* PEI, scrambled miRNA, and miR-29a inhibitor 2 nmol; two-way repeated measures analysis of variance (RM anova) with Bonferroni *post hoc* correction, *n*=9/group. Individual data points are shown alongside bar charts to illustrate data variability and distribution. All data are presented as mean (standard deviation). (c) I.T. injection of miR-29a inhibitor 4 nmol significantly increased IFNR1 expression and reversed the upregulation of p-ERK induced by CFA injection (CFA 5 days group: rats receiving PEI injection 5 days post-CFA). ∗∗∗*P*<0.001, CFA 5 days + miR-29a inhibitor *vs* CFA 5 days; one-way RM anova with Bonferroni *post hoc* correction, *n*=9/group. Individual data points are plotted alongside bars to depict data distribution. All data are presented as mean (standard deviation). Original Western blot images are provided in the Supplementary material. CFA, complete Freund's adjuvant; GADPH, glyceraldehyde-3-phosphate dehydrogenase; miR-29a, microRNA-29a; miRNA, microRNA; p-ERK, phospho-ERK; PEI, polyethyleneimine.

**Fig. S3:**
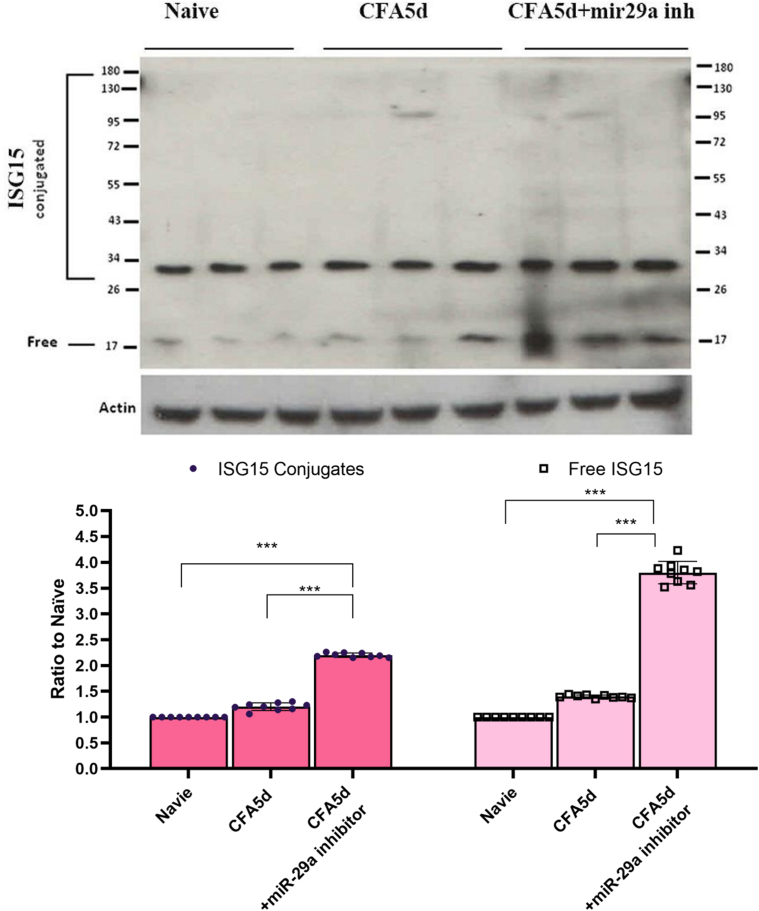


I.t. injection of the miR-29a inhibitor 4 nmol, but not 2 nmol, on the 5th day after CFA injection significantly abrogated the decreases in the mechanical threshold and thermal withdrawal latency for 5 h (Fig. 4a and b). Upregulation of the IFNR1 was noted at 1 h after injection of miR-29a inhibitor 4 nmol ml^−1^ (Fig. 4c). Simultaneously, the upregulation of ISG15 and downregulation of p-ERK were noted (Fig. 4c, Supplementary Fig. S3).

### Examination of the attenuation of miR-29a-induced mechanical hyperalgesia by a p-ERK antagonist in naïve rats

**Fig 5:**
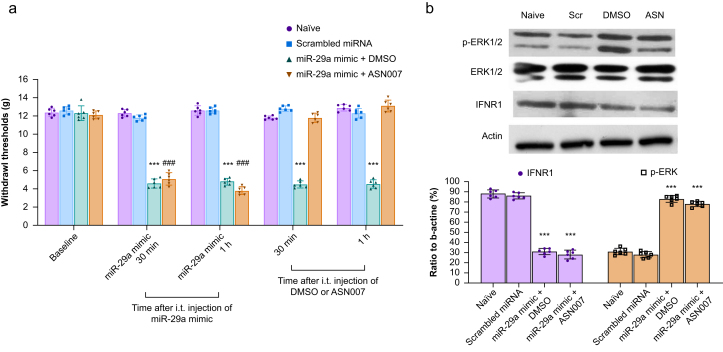
MiR-29a-induced mechanical allodynia in naïve rats is attenuated by p-ERK antagonists. (a) Intrathecal (i.t.) miR-29a mimic-induced mechanical allodynia was significantly attenuated by treatment with the p-ERK antagonist ASN007 30 μg. ∗∗∗*P*<0.001, 30 min and 1 h after i.t. injection of miR-29a mimic (DMSO group) *vs* naïve; ###*P*<0.001, 30 min and 1 h after i.t. injection of miR-29a mimic (ASN007 group) *vs* naïve. ∗∗∗*P*<0.001, 30 min after i.t. injection of miR-29a mimic + DMSO *vs* naïve; 1 h after i.t. injection of miR-29a mimic + ASN007 *vs* naïve; two-way repeated measures analysis of variance (RM anova) with Bonferroni *post hoc* correction, *n*=6/group. Individual data points are shown alongside bar charts to illustrate variability and distribution, providing a clear visual representation of the data. All data are presented as mean (standard deviation). (b) Western blot results show that i.t. injection of the miR-29a mimic significantly increased the expression of p-ERK and decreased the expression of type 1 interferon receptor (IFNR1). ∗∗∗*P*<0.001 for IFNR1: miR-29a mimic + DMSO *vs* naïve, miR-29a mimic + ASN007 *vs* naïve; p-ERK: miR-29a mimic + DMSO *vs* naïve, miR-29a mimic + ASN007 *vs* naïve; one-way RM anova with Bonferroni *post hoc* correction, *n*=6/group. Individual data points are plotted alongside bar charts to depict data variability and distribution. All data are presented as mean (standard deviation). Original Western blot images are provided in the Supplementary material. DMSO, dimethyl sulfoxide; miR-29a, microRNA-29a; miRNA, microRNA; p-ERK, phospho-ERK.

To further confirm the association between p-ERK and miR-29a, we injected the p-ERK antagonist ASN007 30 μg 1 h after the injection the miR-29a mimic (4 nmol). Treatment with ASN007 30 μg reversed the mechanical hyperalgesia induced by the miR-29a mimic for 1 h (two-way RM anova; F(3.208, 64.14)=303.6 for factor time, *P*<0.001; F(3, 20)=1076 for factor group, *P*<0.001; F(12, 80)=189.7 for time × group interaction, *P*<0.001; Fig. 5a). Simultaneously, the downregulation of IFNR1 and upregulation of p-ERK were noted after injection of the miR-29a mimic (Fig. 5b). Thus, these findings further demonstrated that the mechanical hyperalgesia induced by the miR-29a mimic resulted from the upregulation of p-ERK.

## Discussion

The results of this study showed that miR-29a was significantly downregulated on Day 2 and upregulated on Days 3–5 before returning to baseline by Day 7 after CFA injection. IFNR1 were significantly downregulated on Days 3–5 after CFA injection. IFN-β was significantly upregulated from Days 2–10 after CFA injection. I.t. injection of IFN-β attenuated CFA-induced mechanical and thermal hyperalgesia 2 days after CFA injection. However, miR-29a mimic injections blocked the effects of IFN-β. After injecting the miR-29a mimic, IFNR1 were found to be downregulated. ISG15 downregulation and p-ERK upregulation were also observed. Rats injected with IFN-β exhibited significant upregulation of ISG15 and downregulation of p-ERK and these effects were blocked by miR-29a mimics. The attenuation of CFA-induced mechanical and thermal hyperalgesia was observed after i.t. injection of the miR-29a inhibitor. Moreover, after miR-29a inhibitor injection, the expression of IFNR1 and ISG15 increased, and the expression of p-ERK decreased. As a result, IFN-β injection may exert antinociceptive effects, and miR-29a may abrogate these antinociceptive effects by inhibiting the expression of IFNR1. Moreover, miR-29a induced mechanical allodynia in naïve rats. In contrast, an miR-29a inhibitor induced the upregulation of IFNR1 and subsequently exerted antinociceptive effects. These findings collectively indicate that miR-29a modulates IFN-β-induced antinociception and CFA-induced inflammatory pain by inhibiting or inducing the expression of IFNR1. The temporal changes in miR-29a, IFN-β, and IFNR1 expression are involved in the regulation of inflammatory pain.

The changes in the expression of miR-29a, IFN-β, and IFNR1 in the spinal cord after CFA injection were examined over a 10-day period. In Bai and colleagues' study^26^ miR-29a was significantly downregulated within 4 h, significantly upregulated on Day 4, and then returned to its baseline level on the 12th day after CFA injection. Förster and colleagues^12^ have demonstrated that miR-29 was upregulated early after treatment with IFN-β, yet downregulated by 48 h. It has been demonstrated that miR-29a reduces the expression of IFNAR1 in murine thymic epithelial cells, thereby reducing IFN responses.^27^ Hecker and colleagues^11^ revealed that mice lacking miR-29a exhibited increased expression of thymic IFNAR1 and hypersensitivity to polyI:C treatment. The changes in miR-29a expression in this study were similar to those reported in reports by Förster and colleagues^12^ and Bai and colleagues.^26^ Given that miR-29 targets IFNR1, this regulatory relationship may involve a negative feedback loop that inhibits the effect of type 1 IFN, providing inherent protection from the harmful effects of overactivation or inappropriately sustained responses. From 3 to 5 days after CFA injection, IFNR1 was downregulated, while miR-29a was upregulated, suggesting that a negative feedback mechanism limited the progressive increase in IFN-β levels. To our knowledge, this study was the first to demonstrate the changes in the expression of miR-29a, IFN-β, and IFNR1 over time.

It has been reported that IFN-β has various mechanisms that are responsible for its antinociceptive effects.^28^, ^29^, ^30^, ^31^, ^32^ A study by Stokes and colleagues^28^ showed that IFN-β can alleviate tactile allodynia induced by i.t. toll-like receptor 2 (TLR2) or TLR4 ligands. IFN-β transiently relieves nociceptive responses in a murine arthritis model, while the administration of an anti-tumour necrosis factor-α antibody along with IFN-β results in long-lasting relief. The long-term relief may be the result of IL-10 being expressed in the spinal cord of male mice by IFN-β.^29^ In addition, Liu and colleagues^30^ demonstrated that an i.t. injection of IFN-β (1000 or 5000 U) attenuated nerve injury-induced mechanical allodynia for several days in mice without affecting motor activity. IFN-β might exert this long-lasting effect by inhibiting mitogen-activated protein kinase (MAPK) activation, which plays a key role in pain pathogenesis,^31^ and promoting ISG-15 release after nerve injury in mice.^30^ Further evidence of the antinociceptive action of IFN-β can be found in naïve mice that received an i.t. injection of IFN-β.^31^ Moreover, the effects of miR-29a mimics and inhibitors on ISG15 and p-ERK were examined in this study. After miR-29a mimic administration, ISG15 was downregulated, and p-ERK was upregulated. In contrast, miR-29a inhibitors increased ISG15 levels and decreased p-ERK levels. The p-ERK antagonist ASN-007 was able to ameliorate the mechanical allodynia induced by the miR-29a mimic in naïve rats, confirming that this hyperalgesia was related to p-ERK production. Naïve rats injected with the miR-29a mimic exhibited increased p-ERK levels, further corroborating the correlation between mechanical allodynia and p-ERK. This result was consistent with the results reported by Liu and colleagues.^30^

ISGylation is the process by which the ubiquitin-like modifier ISG15 is conjugated to cellular substrate proteins.^33^ This modification can directly regulate IFN signalling and inhibit viral infection. ISGylation involves three key enzymes: the E1 enzyme UBE1L, the E2 enzyme UBCH8, and the E3 ligase HERC5. DeISGylation, the reverse process, is mediated by USP18 (also known as UBP43).^34^ Interestingly, IFN also induces the expression of UBE1L, UBCH8, HERC5, and UBP43. ISG15 exists in two forms: conjugated and free. During ISGylation, ISG15 is covalently attached to target proteins, a modification similar to ubiquitination.^17^ Interferons, immune signalling molecules activated by various challenges, drive this process. Free ISG15, in contrast, remains unbound to other proteins and can function independently within the cell. Primarily produced in response to interferon signalling, free ISG15 can act as a cytokine, being released from cells and binding to receptors on neighbouring cells, thereby triggering an immune response through a signalling cascade.^34^

In this study, both conjugated and free ISG15 were upregulated in rats treated with IFN-β, while both forms were downregulated in rats receiving IFN-β alongside the miR-29a mimic. Conversely, free and conjugated ISG15 were upregulated in rats treated with the miR-29a inhibitor. As Malakhov and colleagues^17^ reported, ISG15 targets key signal transduction regulators, including ERK1, promoting the degradation of these proteins. In dorsal horn neurones, dorsal root ganglion neurones, and epidermal nerve terminals, ERK is activated by peripheral noxious stimuli and inflammation.^35^ Inhibiting ERK activation can reduce both peripheral and central sensitisation, thereby alleviating inflammatory pain, as demonstrated in this study. To further confirm the role of miR-29a in inducing hyperalgesia through p-ERK, we administered the p-ERK inhibitor ASN 007, which attenuated the mechanical allodynia induced by miR-29a mimic administration in naïve rats. Notably, p-ERK was upregulated in the same tissue samples after miR-29a mimic injection, correlating with the development of mechanical allodynia.^36^^,^^37^ Thus, we propose that miR-29a not only induces hyperalgesia but also diminishes the antinociceptive effect of IFN-β on CFA-induced hyperalgesia.

The present study has some limitations. Firstly, as we exclusively used male rats, we were unable to address potential sex differences in the modulation of pain by IFN-β and IFNR1 signalling in rodents. It is known that sexual dimorphism can affect neuroimmunity and pain signaling,^38^ so the findings from this study cannot be directly extrapolated to female rats. In addition, a more in-depth study using knockout mice, such as IFNR1-, ISG15-, and ERK-deficient mice, is needed.

In conclusion, our study illustrated that i.t. administration of IFN-β effectively alleviated CFA-induced mechanical allodynia and thermal hyperalgesia in rats. The introduction of the miR-29a-3p mimic induces both mechanical and thermal hyperalgesia, suppressing IFNR1 expression while downregulating ISG15 and upregulating p-ERK. Conversely, the miR-29a-3p inhibitor inhibited mechanical and thermal hyperalgesia and upregulated the expression of IFNR1 and ISG15, leading to the downregulation of p-ERK. The concerted actions of miR-29a and IFN-β play a crucial role in modulating CFA-induced inflammatory pain by regulating the expression of IFNR1, ISG15, and p-ERK.

This new understanding of the effects of miR-29a and IFN-β and their mechanisms of action opens up novel therapeutic avenues for addressing inflammatory pain. Notably, higher levels of type 1 IFN and elevated levels of homeostatic IFN are expressed in neurones to control viral infections.^39^ Consequently, inducing the release of type 1 IFN in neurones has emerged as a promising potential treatment for pain. However, the experimental nature of our study may not accurately reflect endogenous levels of miR-29a or IFN-ß during inflammation. Therefore, future research should aim to quantify the endogenous concentrations of these agents under physiological and pathophysiological conditions, and assess how they relate to the experimental doses used in this study. Such research will be crucial in determining whether these findings can be translated into therapeutic strategies for managing inflammatory pain. This study provides key mechanistic insights into the role of miR-29a in inflammatory pain, particularly through its regulation of IFNR1. Understanding this interaction could pave the way for new therapeutic strategies in pain management. The findings identify miR-29a as a promising therapeutic target for alleviating inflammatory pain, highlighting its potential to modulate pain pathways effectively. By elucidating the interplay between miR-29a, IFN-ß, and IFNR1, this study lays the groundwork for novel therapeutic interventions targeting these molecular pathways to manage inflammatory pain effectively.

## Authors’ contributions

Conceptualisation: CCL, PHT

Formal analysis: CCL, LHC, EYKH

Investigation: CCL, YYL

Software: CCL

Validation: LHC

Writing—original draft: KCH, CCC, PHT

Writing—review and editing: YYL, EYKH

## Declaration of generative AI and AI-assisted technologies in the writing process

During the preparation of this work the author(s) used ChatGPT 3.5 in order to improve readability and language of the work. After using this tool/service, the author(s) reviewed and edited the content as needed and take(s) full responsibility for the content of the publication.

## References

[1] Kanoh S., Rubin B.K. (2010). Mechanisms of action and clinical application of macrolides as immunomodulatory medications. Clin Microbiol Rev.

[2] Biron C.A. (2001). Interferons alpha and beta as immune regulators--a new look. Immunity.

[3] Pestka S., Langer J.A., Zoon K.C., Samuel C.E. (1987). Interferons and their actions. Annu Rev Biochem.

[4] Platanias L.C. (2005). Mechanisms of type-I- and type-II-interferon-mediated signalling. Nat Rev Immunol.

[5] Owens T., Khorooshi R., Wlodarczyk A., Asgari N. (2014). Interferons in the central nervous system: a few instruments play many tunes. Glia.

[6] Reyes-Vázquez C., Prieto-Gómez B., Dafny N. (2012). Interferon modulates central nervous system function. Brain Res.

[7] Dafny N., Yang P.B. (2005). Interferon and the central nervous system. Eur J Pharmacol.

[8] Dhib-Jalbut S., Marks S. (2010). Interferon-beta mechanisms of action in multiple sclerosis. Neurology.

[9] Di Filippo M., Tozzi A., Tantucci M. (2014). Interferon-β1a protects neurons against mitochondrial toxicity via modulation of STAT1 signaling: electrophysiological evidence. Neurobiol Dis.

[10] Kawashima T., Kosaka A., Yan H. (2013). Double-stranded RNA of intestinal commensal but not pathogenic bacteria triggers production of protective interferon-β. Immunity.

[11] Hecker M., Thamilarasan M., Koczan D. (2013). MicroRNA expression changes during interferon-beta treatment in the peripheral blood of multiple sclerosis patients. Int J Mol Sci.

[12] Forster S.C., Tate M.D., Hertzog P.J. (2015). MicroRNA as type 1 interferon-regulated transcripts and modulators of the innate immune response. Front Immunol.

[13] Milligan E.D., Watkins L.R. (2009). Pathological and protective roles of glia in chronic pain. Nat Rev Neurosci.

[14] Tsuda M., Masuda T., Kitano J., Shimoyama H., Tozaki-Saitoh H., Inoue K. (2009). IFN-gamma receptor signaling mediates spinal microglia activation driving neuropathic pain. Proc Natl Acad Sci U S A.

[15] Liu C.C., Cheng J.T., Li T.Y., Tan P.H. (2017). Integrated analysis of microRNA and mRNA expression profiles in the rat spinal cord under inflammatory pain conditions. Eur J Neurosci.

[16] Zhang D., Zhang D.E. (2011). Interferon-stimulated gene 15 and the protein ISGylation system. J Interferon Cytokine Res.

[17] Malakhov M.P., Kim K.I., Malakhova O.A., Jacobs B.S., Borden E.C., Zhang D.E. (2003). High-throughput immunoblotting. Ubiquitiin-like protein ISG15 modifies key regulators of signal transduction. J Biol Chem.

[18] Zimmermann M. (1983). Ethical guidelines for investigations of experimental pain in conscious animals. Pain.

[19] Kilkenny C., Browne W.J., Cuthill I.C., Emerson M., Altman D.G. (2010). Improving bioscience research reporting: the ARRIVE guidelines for reporting animal research. PLoS Biol.

[20] Maruta T., Nemoto T., Hidaka K. (2019). Upregulation of ERK phosphorylation in rat dorsal root ganglion neurons contributes to oxaliplatin-induced chronic neuropathic pain. PLoS One.

[21] Zhuang Z.Y., Gerner P., Woolf C.J., Ji R.R. (2005). ERK is sequentially activated in neurons, microglia, and astrocytes by spinal nerve ligation and contributes to mechanical allodynia in this neuropathic pain model. Pain.

[22] Chaplan S.R., Bach F.W., Pogrel J.W., Chung J.M., Yaksh T.L. (1994). Quantitative assessment of tactile allodynia in the rat paw. J Neurosci Methods.

[23] Dixon W.J. (1980). Efficient analysis of experimental observations. Annu Rev Pharmacol Toxicol.

[24] Hargreaves K., Dubner R., Brown F., Flores C., Joris J. (1988). A new and sensitive method for measuring thermal nociception in cutaneous hyperalgesia. Pain.

[25] Liu C.C., Gao Y.J., Luo H. (2016). Interferon alpha inhibits spinal cord synaptic and nociceptive transmission via neuronal-glial interactions. Sci Rep.

[26] Bai G., Ambalavanar R., Wei D., Dessem D. (2007). Downregulation of selective microRNAs in trigeminal ganglion neurons following inflammatory muscle pain. Mol Pain.

[27] Papadopoulou A.S., Dooley J., Linterman M.A. (2011). The thymic epithelial microRNA network elevates the threshold for infection-associated thymic involution via miR-29a mediated suppression of the IFN-α receptor. Nat Immunol.

[28] Stokes J.A., Corr M., Yaksh T.L. (2013). Spinal toll-like receptor signaling and nociceptive processing: regulatory balance between TIRAP and TRIF cascades mediated by TNF and IFNβ. Pain.

[29] Woller S.A., Ocheltree C., Wong S.Y. (2019). Neuraxial TNF and IFN-beta co-modulate persistent allodynia in arthritic mice. Brain Behav Immun.

[30] Liu S., Karaganis S., Mo R.F., Li X.X., Wen R.X., Song X.J. (2020). IFNβ treatment inhibits nerve injury-induced mechanical allodynia and MAPK signaling by activating ISG15 in mouse spinal cord. J Pain.

[31] Ji R.R., Gereau R.W.T., Malcangio M., Strichartz G.R. (2009). MAP kinase and pain. Brain Res Rev.

[32] Donnelly C.R., Jiang C., Andriessen A.S. (2021). STING controls nociception via type I interferon signalling in sensory neurons. Nature.

[33] Malakhova O.A., Yan M., Malakhov M.P. (2003). Protein ISGylation modulates the JAK-STAT signaling pathway. Genes Dev.

[34] Skaug B., Chen Z.J. (2010). Emerging role of ISG15 in antiviral immunity. Cell.

[35] Kawasaki Y., Kohno T., Zhuang Z.Y. (2004). Ionotropic and metabotropic receptors, protein kinase A, protein kinase C, and Src contribute to C-fiber-induced ERK activation and cAMP response element-binding protein phosphorylation in dorsal horn neurons, leading to central sensitization. J Neurosci.

[36] Dai Y., Iwata K., Fukuoka T. (2002). Phosphorylation of extracellular signal-regulated kinase in primary afferent neurons by noxious stimuli and its involvement in peripheral sensitization. J Neurosci.

[37] Donnelly C.R., Andriessen A.S., Chen G. (2020). Central nervous system targets: glial cell mechanisms in chronic pain. Neurotherapeutics.

[38] Rosen S., Ham B., Mogil J.S. (2017). Sex differences in neuroimmunity and pain. J Neurosci Res.

[39] Cavanaugh S.E., Holmgren A.M., Rall G.F. (2015). Homeostatic interferon expression in neurons is sufficient for early control of viral infection. J Neuroimmunol.

