# Supplementary material for: The concerted actions of microRNA-29a and interferon-β modulate complete Freund's adjuvant-induced inflammatory pain by regulating the expression of type 1 interferon receptor, interferon-stimulated gene 15, and p-extracellular signal-regulated kinase
Source: BJA Open. 2025 Feb 3;13:100376. doi: 10.1016/j.bjao.2024.100376 (PMC11840201; doi:10.1016/j.bjao.2024.100376)
Supplement: Multimedia component 1 [file mmc1.docx]

Legends for supplementary figures

**Supplementary Fig. 1 Temporal changes in IFN-β and IFNR1 expression after CFA injection.** (B) A significant increase in IFN-β levels was observed starting on the 2nd day post-CFA injection. Individual data points are shown along with bars representing group means. *p = 0.01, **p = 0.002, ***p < 0.001 vs. naïve; one-way RM ANOVA with Bonferroni post hoc correction; n = 6/group. IFNR1 expression decreased significantly on the 3rd and 5th days after CFA injection. Individual values are displayed with bars representing the group means for clarity. ##p = 0.004 vs. naïve; one-way RM ANOVA with Bonferroni post hoc correction; n = 6/group. Data are presented as mean ± SD.

**Supplementary Fig. 2. Expression of conjugate and free interferon-stimulating gene 15 (ISG15) in the spinal cord of CFA-injected rats following intrathecal (i.t.) injection of interferon-β with or without 4 nmol miR-29a mimic.**
i.t. injections of 10000 U IFN-β significantly increased the expression of both conjugate and free ISG15. ***p < 0.001, CFA 2d + IFN-β vs. CFA 2d. Co-administration of 4 nmol miR-29a mimic with IFN-β inhibited the upregulation of conjugate and free ISG15 induced by 10000 U IFN-β. ###p < 0.001, CFA 2d + IFN-β + miR-29a mimic vs. CFA 2d + IFN-β; one-way RM ANOVA with Bonferroni post hoc correction, n = 8/group. Individual data points are plotted alongside bar charts to demonstrate the variability and distribution of the data. All data are presented as mean ± SD.

**Supplementary Fig. 3. Expression of conjugate and free ISG15 in the spinal cord of rats following intrathecal (i.t.) injection of 4 nmol miR-29a inhibitor five days after CFA injection.**
**i.t.** injection of 4 nmol miR-29a inhibitor significantly increased the expression of free ISG15. ***p < 0.001, CFA 5d + miR-29a inhibitor vs. CFA 5d; one-way RM ANOVA with Bonferroni post hoc correction, n = 9/group. Individual data points are displayed alongside bar charts to show data variability and distribution. All data are presented as mean ± SD.
